# Determination of PAH Contamination in Breast Milk Samples from Hungarian Volunteering Mothers, Using HPLC–FLD

**DOI:** 10.3390/molecules29215060

**Published:** 2024-10-26

**Authors:** Bernard Collins Anditi, Viktória Poór, Dénes Szerencsés, István Szabó, Mátyás Wahr, Anikó Kőnig-Péter, Timea Dergez

**Affiliations:** 1 Doctoral School of Chemistry, Faculty of Sciences, University of Pécs, 7624 Pécs, Hungary; collinsanditi2@gmail.com (B.C.A.);; 2 Institute of Bioanalysis, Medical School, University of Pécs, 7624 Pécs, Hungary; viktoria.poor@aok.pte.hu (V.P.); aniko.konig@aok.pte.hu (A.K.-P.); 3 Department of Environmental Toxicology, Hungarian University of Agricultural and Life Sciences, 2100 Gödöllő, Hungary; szabo.istvan.temi@uni-mate.hu

**Keywords:** polycyclic aromatic hydrocarbon (PAH), HPLC–FLD, breast milk, total PAHs, incremental life risk calculation (IRCL)

## Abstract

(1) The evidence is mounting that polycyclic aromatic hydrocarbons (PAHs) are a class of hazardous organic compounds with established carcinogenic and toxic properties. Humans may be exposed to PAHs through several different routes, including diet, inhalation, and dermal contact. There is also a possibility that they could transfer into breast milk following maternal exposure, which could potentially endanger breastfeeding infants. (2) The objective of this study was to ascertain the concentration of polycyclic aromatic hydrocarbons (PAHs) in breast milk samples from 50 Hungarian mothers, employing high-performance liquid chromatography with fluorescence detection (HPLC–FLD). An Incremental Life Risk Calculation (IRCL) model estimated the carcinogenic risk to infants. (3) Total PAH concentrations ranged from 0 to 78 ng/mL, with fluorene (5.3 ng/mL), phenanthrene (3.2 ng/mL), and pyrene (2.5 ng/mL) being the most abundant. PAHs were detected in 48 of the 50 samples, with phenanthrene present in 92% of samples. Dibenzo (a,h)anthracene was not detected. (4) According to the model measurements, most of the samples were within acceptable risk levels; however, 2 samples out of 50 posed a higher risk. Statistical analysis of questionnaires completed by the mothers indicated that factors such as diet, residence, and education may influence PAH levels in breast milk.

## 1. Introduction

Polycyclic aromatic hydrocarbons (PAHs) are formed as a consequence of incomplete combustion of organic materials, including coal, oil, gas, wood, tobacco, charbroiled meats, garbage, and other organic substances. [1,2,3]. They are released from natural and mainly anthropogenic sources [4].

PAHs can mean health concerns due to their probable carcinogenic, mutagenic, and teratogenic effects in humans [5,6]. The degree of toxicity, carcinogenicity, and mutagenicity depends on the type of PAH. The heavy PAHs (containing 4–6 rings) are more toxic and stable compared to light ones (2–3 rings) [7] (Table 1). The U.S. Environmental Protection Agency (US EPA, 1993) and the International Agency for Research on Cancer (IARC) [6,8] have classified polycyclic aromatic hydrocarbons (PAHs) according to their carcinogenic potential, as illustrated in Table 1. Furthermore, some PAHs that are not considered carcinogenic have been observed to exert effects on the immune system and endocrine regulation [9].

As illustrated in Table 1, PAHs are classified according to their molecular weight (MW), with low-MW (2–3 rings) and high-MW (4–6 rings) categories distinguished. The US EPA employs a classification system for PAHs that is based on their carcinogenic potential. Group B2 encompasses substances designated as "possibly carcinogenic to humans," whereas Group D is "unclassifiable as to carcinogenicity." The classification comprises the following categories: The classification is as follows: 1 (carcinogenic), 2A (probable carcinogenic), 2B (possible carcinogenic), and 3 (not classifiable). Toxic Equivalency Factors (TEFs) are employed to express the toxicity of PAHs in terms of their carcinogenic potential, with benzo(a)pyrene (BaP) serving as a reference point [10]. BaP is regarded as the most toxic and carcinogenic PAH [11,12]. The Contam panel [8] has identified PAH4 (the sum of benzo(a)anthracene, chrysene, benzo(b)fluoranthene, and BaP) as the optimal indicator of PAH occurrence in food. The European Commission has established maximum permissible levels in oils and fats, with limits of 2 μg/kg for BaP and 10 μg/kg for PAH4 [13]. While the human body is capable of metabolizing and eliminating PAHs [13,14], continuous exposure through diet and atmospheric contamination may result in pseudo-persistence [15,16].

These compounds can accumulate in lipophilic regions such as adipose tissue, liver, and kidney. PAHs may be excreted in breast milk because of their apolar character [17]. Breast milk is further susceptible to accumulating PAHs as it contains lipophilic tissue [18]. The occurrence of contamination is contingent upon a few factors, including the environmental context, the source of exposure, the stage of lactation, the medical status of the individual, the rearing system, and the lifestyle of the subject [19,20].

There is evidence to suggest that chronic exposure during the periods of motherhood and early childhood can have long-term consequences including birth defects, premature birth, low birth weight, malformations [21], reduced growth and development [22], asthma [23], and lower intelligence quotient (IQ) [21].

PAHs-contaminated breastmilk has been reported in numerous studies all over the world, including Europe, Asia, America, and Africa: Europe: Italy [13,19,24], Portugal [25], Czech republic [26]; Asia: Turkey [27], China [28], Hong Kong [29], India [30]; America: [15,31,32]; and Africa: Egypt [18], Ghana [33], Uganda [34]. 

The present study aims to assess the levels of PAHs in the breast milk of healthy Hungarian women and to perform a carcinogenic risk assessment to determine whether levels of PAHs in breast milk pose any risk to breastfed infants. We aim to further reveal factors that can affect the PAH concentrations in breast milk. To the best of our knowledge, this is the inaugural study to examine the concentration of PAHs in the breast milk of Hungarian mothers.

## 2. Results

### 2.1. Identification of PAHs by HPLC–FLD

**Figure 1:**
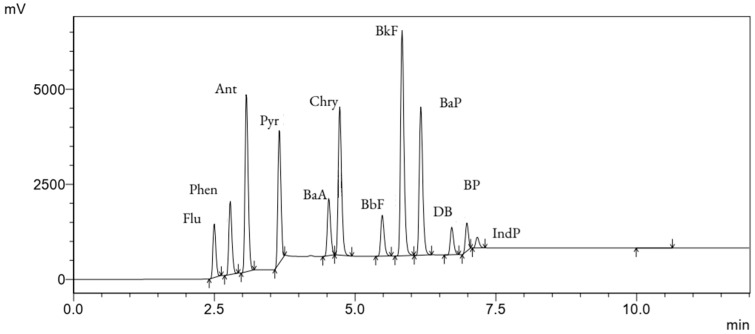
Chromatogram of the PAH standard solution, illustrating retention times for specific PAHs used in the analysis of breast milk samples. Peaks corresponding to individual PAHs are marked, enabling their differentiation. The analysis was conducted using HPLC–FLD.

The Figure 1 shows the chromatogram of the PAH standard solution with retention times, which was subsequently used for the determination of PAHs in the breast milk samples. The identified peaks correspond to specific PAHs, facilitating their differentiation. Table 2 contains the PAH compounds along with their corresponding retention times. 

Detailed validation data are presented in Table 2 with LOD and LOQ, linearity, and the mean recovery rate in the case of 12 PAHs.

### 2.2. PAH Profile of Breast Milk Samples and Exposure Assessment

The concentrations of twelve PAHs were determined in the breast milk of 50 Hungarian mothers. The sum of all individually measured components and four PAHs were also calculated in each sample and indicated as four PAHs and total PAHs, respectively.

Out of the 50 samples analyzed, 48 showed the presence of at least two PAHs; however, in the other two samples, no PAHs were detected. Phenanthrene appeared most frequently (found in 92% of the samples). Dibenzo (a.h) anthracene was not detected in any of the analyzed samples. Fluorene was the most abundant PAH (5.3 ng/mL), followed by phenanthrene (3.2 ng/mL) and pyrene (2.5 ng/mL). In total, 10 components were found in the sample, which contained the most different kinds of PAHs. The concentrations of four PAHs ranged from ND to 13.6 ng/mL, with a mean concentration of 4.4 ng/mL (Table 3).

The most frequently detected PAH (phenanthrene) and the most abundant PAH (fluorene) were also low-molecular-weight PAH compounds, which may be transferred in human breast milk most rapidly and in greater amounts, due to the inverse relationship between molecular weight and mammary transport. [35].

The total PAHs in human breast milk exhibited a range of 0 to 77.95 ng/mL, with a mean value of 17.87 ng/mL. Mean concentrations of individual PAHs in the breast milk samples of Hungarian women fell within the range of 0–6 ng/mL. 

The analysis of PAH levels in the present study reveals that the detected concentrations are within a comparable range to those reported in previous research conducted in European countries, specifically Italy and the Czech Republic. This finding is consistent with previous publications [19,26], indicating a similar exposure pattern across these regions. The comparable PAH levels suggest that common sources and exposure pathways may exist, potentially related to similar environmental pollution, dietary habits, or lifestyle factors prevalent in these areas.

There is a significant trend observed consisting of higher mean levels of low-molecular-weight (LMW) PAHs compared to heavy PAHs. This trend aligns with findings of previous studies, where LMW PAHs, such as naphthalene and phenanthrene, were more prevalent in breast milk than heavier compounds like benzo(a)pyrene. The higher concentration of LMW PAHs may be attributed to their greater volatility and higher abundance in the environment, which facilitates their absorption and transfer into breast milk. Additionally, these compounds are often found in higher concentrations in urban areas due to traffic emissions and residential heating, which are common sources of LMW PAHs.

The consistency of these results across different geographic locations underscores the importance of monitoring PAH levels in human breast milk as a marker of environmental exposure. It also highlights the need for public health interventions aimed at reducing exposure to these hazardous compounds, particularly for breastfeeding infants who are among the most vulnerable populations. Future research should focus on identifying specific sources of PAHs in these regions, as well as examining the potential long-term health effects on infants exposed to these contaminants through breastfeeding. Such efforts are crucial for developing effective strategies to minimize exposure and protect public health.

### 2.3. Risk Assessment

ILCR values were calculated to estimate carcinogenic risk for infants. Out of the fifty samples, five had ILCR values less than 10^−6^, which is considered safe. Only two of the samples had an ILCR value higher than 10^−4^, indicating a potential carcinogenic risk. Understandably, this milk sample showed a high ILCR value because it came from one of three mothers who smoked tobacco. 

This sample was additionally characterized by the following factors: frequent consumption of meat, lack of organic products in the diet, no higher education qualifications, and living in an urban setting. All these factors may collectively indicate a higher risk level.

The remaining 44 samples exhibited ILCR values within the range of 10⁻⁶ and 10⁻⁴, which is generally considered an acceptable range for infants who are being breastfed.

The mean values for BaP-MOE and PAH4-MOE were found to be 4553.66 and 1,205,083.09, respectively. In the case of BaP, the lowest recorded value was 1112.11, while the highest was 11,131.11. Concerning PAH4-MOE, the lowest value is 1869.55, while the highest value is 22,077,922.08. In the case of BaP-MOE, 19 samples were identified as potentially hazardous, while in the case of PAH4-MOE, 23 possibly dangerous milk samples were identified among the 50 samples. As can be observed, the mean MOE-BaP value is less than 10,000, which suggests the possibility of a potential hazard associated with these human milk samples. It should be noted that the magnitude of MOE provides an indication of the level of concern but is not a precise quantification of risk. In other words, a larger MOE does not necessarily indicate a smaller potential risk associated with target compound exposure. For instance, an MOE of 1000 does not imply that the cancer risk was 10 times higher than that of an MOE of 10,000 [36].

### 2.4. Questionnaires

Several factors can influence the levels of PAHs in breast milk. These factors include the geographical location, residence, diet, smoking habits, and lifestyle of the individuals. 

Various parameters provided by mothers in the questionnaires were analyzed. We hypothesized that polycyclic aromatic hydrocarbon (PAH) compounds would occur in higher quantities in women with a higher BMI due to their lipophilic properties. However, we failed to confirm this outcome. Maternal BMI before and after pregnancy did not correlate with the total PAH concentration (*p*_before_ = 0.262, *p*_after_ = 0.054). 

Environmental pollution may vary in different geographical areas, contributing to higher concentrations of PAHs in the air, water, soil, and food sources. Fortunately, only three mothers smoked during breastfeeding, which made statistical comparisons and conclusions unfeasible. However, the average PAH concentration was still higher in smokers compared to non-smokers, based on the data from these three mothers (smoker: 18.00 ± 17.84 and non-smoker: 17.10 ± 11.65). 

**Figure 2:**
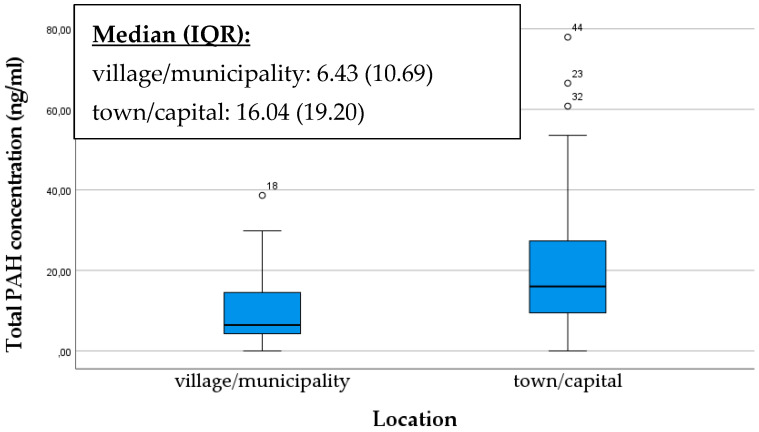
Comparison of total PAH concentration in breast milk samples based on location as village/municipality or town/capital. The data are presented as median and interquartile range (IQR) values. An “◦” indicates an outlier.

Industrial activities, vehicle emissions, and the proximity of contaminated areas can also increase the presence of PAHs in the living environment. Individuals living in areas with higher pollution levels (such as in the capital or major cities, near busy roads or factories) may face an increased risk of exposure to PAHs, which can later affect the PAH levels in breast milk. Accordingly, the objective of our analysis was to ascertain whether a notable discrepancy existed in the overall quantity of PAHs present in the breast milk samples of mothers living in two different areas categorized as village/municipality and town/capital. Our results confirmed that samples from mothers living in urban environments contained significantly more PAHs. The box plot illustrates that outstandingly high levels of PAHs are also present in mothers living in urban environments (*p* < 0.001) (Figure 2).

**Figure 3:**
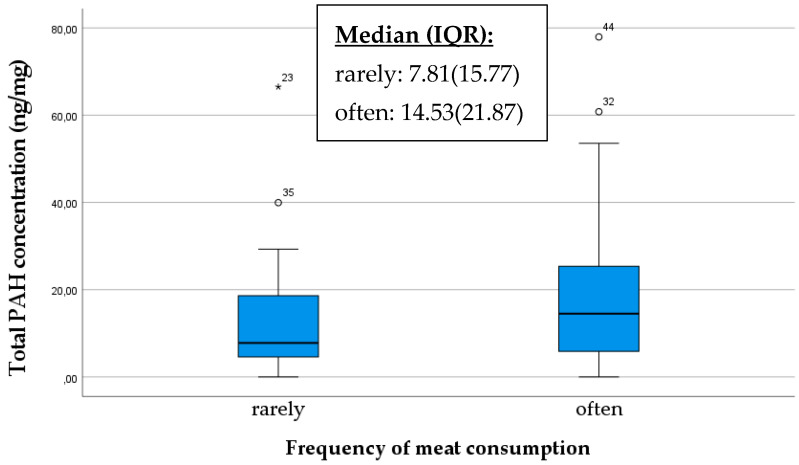
Comparison of total PAH concentration in breast milk samples based on meat consumption as rarely/often. The data are presented as median and interquartile range (IQR) values. An “◦” indicates an outlier and “*” indicates the presence of extreme outliers.

Furthermore, our findings substantiate the hypothesis that dietary habits can influence the concentration of polycyclic aromatic hydrocarbons (PAHs) in breast milk, including meat, dairy products, and organic products potentially affecting the overall PAH concentration. If someone consumes meat or milk contaminated with PAHs, these compounds can enter the bloodstream and subsequently be excreted into various body fluids, including breast milk [34]. Consequently, the PAH concentration in breast milk may increase with higher meat or milk consumption, especially if the meat is cooked at high temperatures or exposed to significant levels of smoke or charring. Based on our sample, mothers who consumed meat more frequently had higher PAH concentrations in their breast milk compared to those who consumed it less (*p* = 0.044) (Figure 3).

**Figure 4:**
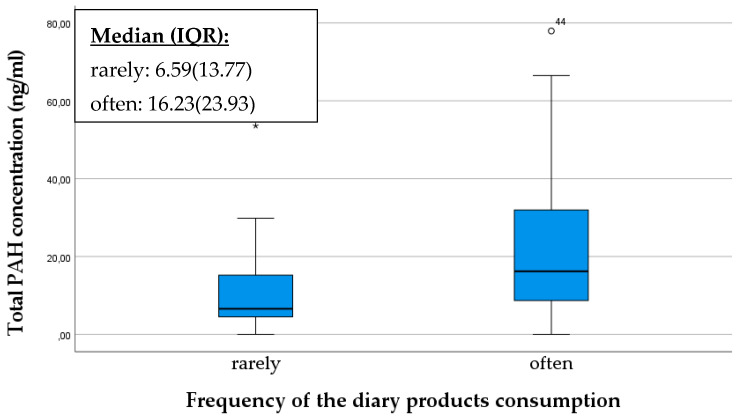
Comparison of total PAH concentration in breast milk, based on the frequency of consumption of dairy products of mothers. The data are presented as median and interquartile range (IQR) values. An “◦” indicates an outlier and “*” indicates the presence of extreme outliers.

The frequency of maternal consumption of dairy products and its influence on the PAH content of breast milk was investigated with curiosity. We obtained the result that individuals who frequently consume dairy products had a significantly higher PAH concentration (*p* = 0.027) (Figure 4). PAHs are typically lipophilic (fat-soluble) compounds, and as such, they can easily accumulate in fatty tissues and animal fats. If foods, such as cow milk, contain PAHs, they can be stored in the body when dissolved in fats [37].

When mothers consume dairy products, the fat content of milk provides an opportunity for the accumulation of PAHs in the body, which can then be detectable in breast milk. This is particularly noteworthy when mothers frequently consume dairy products, as the processing methods of these products, such as pasteurization or heat treatment, may potentially contribute to the formation of PAHs [30].

**Figure 5:**
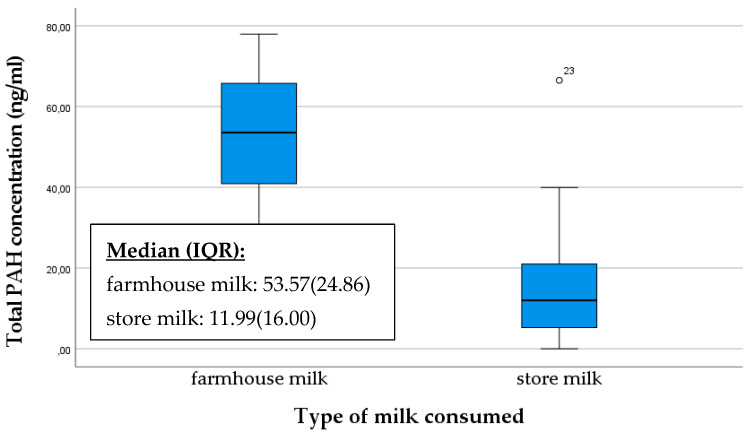
Comparison of total PAH concentration in breast milk samples based on the type of milk consumed. An “◦” indicates an outlier.

We also asked mothers about their milk consumption habits, specifically whether they consume store-bought milk or home-produced milk. The ratio of mothers consuming home-produced milk versus store-bought milk varied significantly, with a total of ten consuming home-produced milk and 33 consuming store-bought milk. The breast milk of mothers who consume home-produced milk had a significantly higher PAH concentration than those consuming store-bought milk (*p* = 0.007) (Figure 5).

The presence of PAHs in dairy products may therefore depend on the processing and preparation methods of the milk. The farmhouse milk samples may have exhibited PAH levels that were higher than those detected in pasteurized milk. This is potentially attributable to the higher triglyceride content in raw milk, which may serve to explain the elevated PAH levels [38].

Processing methods, the source of the milk, and storage conditions can vary for home-produced milk, all of which may influence the quantity of PAHs. Factors such as the diet and living conditions of the animals may also play a role. Store-bought dairy products are also processed and produced in various ways, and their PAH levels are regularly monitored to comply with standards.

Organic farming methods prioritize soil and air purity, thereby minimizing the input of chemical substances such as synthetic pesticides that may contain polycyclic aromatic hydrocarbons (PAHs) or contribute to their formation. Individuals can reduce their exposure to PAHs originating from pesticide residues by consuming organic products. The production of organic food may involve different processing methods compared to traditional foods. Producers of organic food may employ alternative processing techniques that could result in potentially lower PAH levels. In our study, we assessed how the total PAH concentration in the breast milk of mothers would differ between mothers who frequently consumed organic products compared to those that do not [39,40].

**Figure 6:**
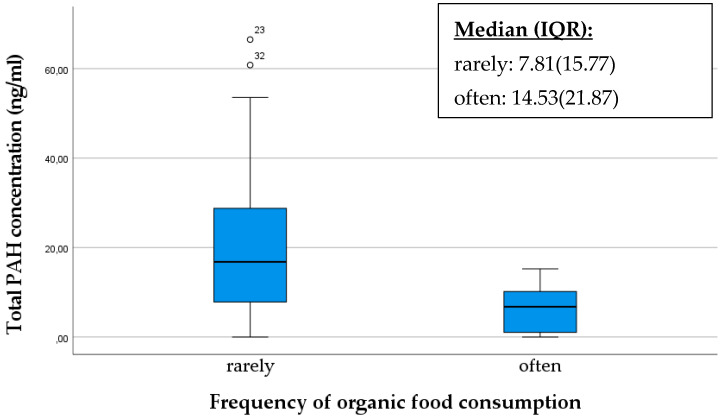
Comparison of total PAH concentration in breast milk samples based on the frequency of organic food consumption as rarely/often. The data are presented as median and interquartile range (IQR) values. An “◦” indicates an outlier.

Our results indicate that mothers who preferentially consumed organic products had a significantly lower PAH concentration as shown in the box plot below (*p* < 0.001). Mothers who infrequently consumed organic products exhibited more pronounced outliers with higher PAH levels (Figure 6).

**Figure 7:**
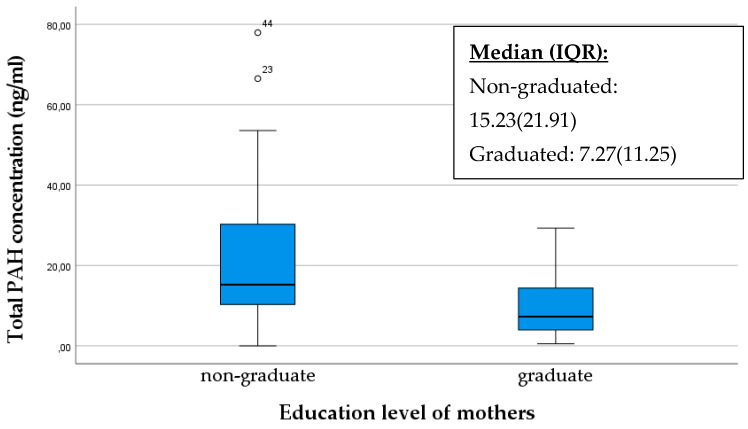
Comparison of the total PAH concentration in breast milk samples based on the education level of mothers. The data are presented as median and interquartile range (IQR) values. An “◦” indicates an outlier.

It is conceivable that educated or better-informed mothers possess more knowledge about potential risks affecting infants, including exposure to pollutants such as polycyclic aromatic hydrocarbons (PAHs). Consequently, they are more likely to adopt healthier lifestyles and make decisions that minimize exposure to harmful substances. The box plot below also illustrates that extremely high PAH values occurred among mothers without a diploma, and their median value was significantly higher (*p* = 0.005) (Figure 7).

## 3. Materials and Methods

### 3.1. Human Subjects

A total of 50 volunteering lactating mothers, randomly selected from the Hungarian population, were included in our study. The recruitment of potential donors was carried out with the help of the Hungarian Nursing Association (Magyar Védőnői Szolgálat). All volunteers were over 18 years old. Participating mothers signed an informed consent form and completed a questionnaire to provide information about their education, residence, dietary habits, Body Mass Index (BMI), smoking, and other relevant information that can influence the levels of PAHs in their breast milk. Further information about the donors can be found in Table 4. 

The sampling of breast milk took place between 2014 and 2019. The breast milk samples were collected manually by the mothers. Approximately 10–20 mL from each participating mother were expressed and placed into clean 50 mL FALCON tubes, were frozen immediately, and stored at −20 °C until the analysis. PAH measurements were performed at the Institute of Bioanalysis at the University of Pécs.

### 3.2. Chemicals and Reagents

The EPA 525 PAH standard mix (500 μg/mL in dichloromethane) contained 13 PAHs acenaphthylene (Ace), anthracene (Ant), benzo(a)anthracene (BaA), benzo(a)pyrene (BaP), benzo(b)fluoranthene (BbF), benzo(g,h,i)perylene (BP), benzo(k)fluoranthene (BkF), chrysene (Chry), dibenzo(a,h)anthracene (DB), fluorene (Flu), indeno(1,2,3-c,d)pyrene (IndP) phenanthrene (Phen), and pyrene (Pyr) was purchased from Sigma Aldrich, (St. Louis, MO, USA).

### 3.3. Sample Preparation and Chemical Analysis

The sample preparation included liquid–liquid extraction of PAHs from breast milk, followed by silica gel purification and concentration steps. Just before the analysis, the breast milk samples were thawed in the dark in a water bath at 37 °C for 10 min. An amount of 5 mL of breast milk was taken into a polypropylene centrifuge tube and mixed with 3 mL of hexane. The solution was mixed vigorously for 5 min, then incubated for 30 min in a water bath at 37 °C, and finally centrifuged at 3000 rpm for 10 min at 4 °C. The liquid above the sediment was removed and transferred for additional purification. A Pasteur pipette was filled with 2 g of silica gel and conditioned with 3 mL of 3:1 n-hexane: dichloromethane (3:1, *v*/*v*) and then washed with 3 mL of hexane; the whole volume of the supernatant was put onto the silica column. The column was washed again with 3 mL of hexane, and then the PAHs were eluted with 3 mL of n-hexane: dichloromethane (3:1, *v/v*). The extract was evaporated to dryness by a gentle stream of nitrogen, redissolved in 1 mL of acetonitrile, and then transferred into dark glass HPLC vials for HPLC–FLD analysis. PAHs were extracted from breast milk in duplicates. The distilled water was used as a method blank and subjected to the entire analytical procedures to determine possible background interference. 

All organic solvents, such as hexane, cyclohexane, dichloromethane, and acetonitrile, that were used for the preparation and analysis of samples were LC-MS grade (VWR International, Radnor, PA, USA).

### 3.4. Instrumental Analysis

The HPLC–FLD separation was performed using a Shimadzu LC 20 AD model HPLC (Shimadzu corp., Kyoto, Japan) apparatus, which included a binary solvent delivery system, an autosampler, a column heater, an RF-20A/RF-20Axs Fluorescence Detector (FLD) (Hamamatsu Corp., Sizuoke, Japan), and a UV diode array detector (DAD) (Hamamatsu corp., Sizuoke, Japan). The separation of PAHs was carried out on a PAH Kinetex column (4.6 mm × 100 mm, 3.5 μm) by Phenomenex (Torrance, California, United States). The sample volume injected was 10 μL. PAHs were separated using a gradient elution program with a flow rate of 1 mL/min. The initial mobile phase was 50% acetonitrile and 50% water for 7 min, which was then gradually changed to 100% acetonitrile over 8 min, and then decreased to the initial phase (50–0%) over 12 min. The column temperature was maintained at 30 °C during the analysis. 

The PAHs were monitored using the FLD. Due to the lack of fluorescent properties of acenaphthylene, no quantitative determination was made in the case of this compound. The excitation and emission wavelengths were changed during the run to obtain the highest sensitivity for all compounds. The effluents were monitored using excitation and emission (Ex/Em) wavelengths: 280/330 nm for Flu; 246/370 nm for Phen; 250/406 nm for Ant; 270/390 nm for Pyr; 265/380 nm for BaA and Chry; 290/430 nm for BbF, BkF, and BaP; 290/450 nm for DB and BP; and 300/500 nm for IndP. PAHs were identified based on retention time and fluorescence spectrum, and compound quantification was performed by using an external calibration method. The HPLC analysis of the samples was repeated in triplicate.

### 3.5. Method Validation

Different concentrations of standard calibration solutions were prepared at nine concentration levels in acetonitrile. The calibration levels were as follows: 0.0625, 0.125, 0.25, 0.5, 1, 5, 10, 20, and 50 ng/mL PAHs. For each level, three independent replicates were prepared in a random order. Spiked samples (final concentrations: 5, 10, and 20 ng/mL) were used for recovery studies. The limit of detection (LOD) and limit of quantitation (LOQ) were calculated based on a signal-to-noise ratio of 3:1 and 10:1, respectively. All solutions were stored in amber vials at 4 °C.

All 12 PAHs have a good linearity at 9 concentrations (0.0625, 0.125, 0.25, 0.5, 1, 5, 10, 20, and 50 ng/mL) with a regression coefficient greater than 0.9870. The LOD and LOQ of the method ranged from 0.07 to 0.25 ng/mL and 0.21–0.76 ng/mL, respectively. The method performed well in the recovery of PAHs in the mothers’ milk both at low (5 ng/mL), medium (10 ng/mL), and high (20 ng/mL) concentrations. The mean recovery rates ranged from 72.81 to 135.08% (Table 2).

### 3.6. Risk Assessment Analysis

The assessment of the potential health risks associated with exposure to PAHs in breast milk is a crucial aspect of this study, especially in the context of the carcinogenic risk to infants. In this section, we present a detailed risk assessment using well-established methods, including Toxicity-Equivalent Factors (TEFs) and the Incremental Lifetime Cancer Risk (ILCR) models. These models allow a comprehensive assessment of the carcinogenic potential of PAHs by converting individual compound concentrations into BaP equivalents and estimating the lifetime cancer risk associated with PAH exposure through breastfeeding.

#### 3.6.1. Exposure Assessment

Toxicity-Equivalent Factors (TEFs) [8] have been defined as carcinogenic or probable carcinogenic PAHs. TEF values represent the relative toxicity of individual PAH compounds compared to a reference compound, which is generally applied to the most toxic, carcinogenic, and mutagenic PAH specifically BaP. The TEF value of BaP is 1. TEF values of other PAHs are shown in Table 1. PEQ-BaP is BaP-equivalent toxicity calculated by the sum of individual PAH concentrations (Ci) multiplied by its corresponding TEF (TEFi) as follows:

PEQBaP = Σ (Ci × TEFi)(1)

The chronic daily intake (CDI) of PAHs was calculated using the BaP-equivalent concentration, as shown in the following equation:

CDI (ng PEQBaP/kg bw d) = Σ(C_i_ × IR_i_ × ED × EF)/(BW × AT) (2)

where C_i_ is the PAH12 (the PAH13-standard mix without acenaphthylene calculated the BaP-equivalent concentration (ng PEQBaP ng/g). IR is the ingestion rate of breast milk in grams per day (700 g milk/day); EF is the exposure frequency to carcinogenic or mutagenic PAHs in days per year (350 days/year); ED is the exposure duration in years (1 year); BW is the average body weight of a baby in kg (5kg); and AT is the average exposure time in days (2 years). The default values were made in consistent US EPA guidelines)

#### 3.6.2. Risk Characterization

Estimating the infant risk for cancer-related PAH content in breast milk can involve the use of various models. We used the Incremental Lifetime Cancer Risk (ILCR) model to calculate carcinogenic risk for infants. The ILCR method is widely used in the risk assessment of PAH [41] as follows:

ILCR = CDI × SF_BaP_ × CF (3)

SF_BaP_ is the carcinogenic slope factor of BaP (7.3 mg/kg), and CF is the unit transformation 10^−6^ mg/ng.

According to the USEPA guidelines, an ILCR lower than 10^−6^ is a safe value, an IRCL in the range 10^−6^ and 10^−4^ is acceptable, and an IRCL higher than 10^−4^ indicates risk for infants [42].

A total of 50 breast milk samples were subjected to analysis, with the ILCR values observed to range from 1.94 × 10^−6^ to 1.95 × 10^−3^. In two instances, the ILCR value exceeded 10^−4^ (1.79 × 10^−3^ and 1.95 × 10^−3^), indicating a potential carcinogenic risk for infants. These findings indicate that, although the majority of samples were within acceptable risk levels, a small percentage posed a higher risk.

To estimate the risk of exposure to BaP and ∑PAH4, the margin of exposure (MOE) was employed using the calculated CDI and the benchmark dose lower confidence limit for BaP and PAH4 (BMDL_10_, 0.07 and 0.34 mg/kg/day). The following equation was used:

(4) $$MOE=\frac{BMDL_{10}}{CDI}$$

### 3.7. Statistical Analysis

Statistical analysis was conducted using IBM SPSS Statistics 29 software (IBM Corporation, Armonk, NY, USA) to find correlations between maternal dietary habits, lifestyle, and the PAH concentrations in breast milk. A descriptive statistical analysis was performed. Data (including maternal demographic characteristics and PAH concentrations) are presented as mean ± standard deviation or as median with interquartile range (IQR), depending on the data distribution. The sample distribution of PAH concentrations was examined before statistical analysis using a Kolmogorov–Smirnov test. The datasets followed a normal distribution in the case of BaA, BbF, and BkF, while in the case of other PAHs, total PAH concentration did not follow the normal distribution. Hypotheses regarding the equality of total PAH concentrations were examined using non-parametric procedures. A Mann–Whitney U-test was used to compare two different groups, while a Kruskal–Wallis test was used for more than two groups. Statistical significance was determined at a threshold of *p* < 0.05.

## 4. Conclusions

The analysis has confirmed the presence of Polycyclic Aromatic Hydrocarbons (PAHs) in breast milk samples obtained from volunteering mothers in Hungary. The average concentration of PAHs in breast milk aligns with the average levels observed in comparable European studies. According to the calculations made with the help of the IRCL model, most breast milk samples can be considered safe or show acceptable carcinogenic risk for infants (between 10% and 88%). The MOE analysis for BaP and PAH4 indicates a potential risk in several human milk samples, as the MOE value for BaP and PAH4 falls below the safety threshold of 10,000. While a higher MOE does not necessarily imply a lower risk, these findings highlight the necessity for careful monitoring of PAH exposure in breastfeeding mothers. Despite this concern, the benefits of human milk for infant development remain significant; however, it is of paramount importance to minimize PAH exposure through dietary and environmental precautions. Monitoring of PAH levels during lactation is recommended to follow potential changes and challenges of infant health risks. The results of this study suggest that lifestyle and dietary habits can play a significant role in regulating the concentration of PAHs in breast milk. Informing mothers about ways to avoid PAH-affected activities (e.g., smoking) and food types (e.g., smoked meat) could also be important. Based on the findings, adopting a healthy lifestyle and a mindful choice of food may contribute to a minimal quantity of PAHs in the diet, which is crucial for proper maternal and infant health. Further research should thoroughly investigate the impact of individual foods, processing methods, and other lifestyle factors on PAH concentration. Healthcare advice should emphasize the role of healthy nutrition and lifestyle in minimizing the presence of PAHs in breast milk.

## Figures and Tables

**Table 1:** Classification of 13 EPA PAHs according to molecular weight and carcinogenicity.

Serial Number	Name of the PAH	Abbreviation	MW	US EPA	IARC	TEF
1	Acenaphtylene	Acy	l	D	3	0.001
2	Fluorene	Flu	l	N/A	3	0.001
3	Phenantrene	Phen	l	D	3	0.001
4	Anthracene	Ant	l	D	3	0.01
5	Pyrene	Pyr	h	D	3	0.001
6	Benzo(a)anthracene	BaA	h	B2	2B	0.1
7	Chrysene	Chry	h	B2	2B	0.01
8	Benzo(b)fluoranthene	BbF	h	B2	2B	0.1
9	Benzo(k)fluoranthene	BkF	h	B2	2B	0.1
10	Benzo(a)pyrene	BaP	h	B2	1	1
11	Dibenzo(a.h)anthracene	DB	h	B2	2A	1
12	Benzo(g,h,i)perylene	BP	h	D	3	0.01
13	Indeno(1.2.3-c.d)pyrene	IndP	h	B2	2B	0.1

**Table 2:** Precision, linearity, detection, and quantification limits of analytical method for PAHs.

PAH	Retention Time (min)	LOD (ng/mL)	LOQ (ng/mL)	Linearity (R^2^)	The Mean Recovery Rate (%)
Fluorene	2.494	0.18	0.55	0.9920	103.60
Phenantrene	2.778	0.21	0.63	0.9912	73.10
Anthracene	3.066	0.14	0.42	0.9961	84.70
Pyrene	3.650	0.25	0.76	0.9873	85.00
Benzo(a)anthracene	4.529	0.10	0.30	0.9980	79.50
Chrysene	4.723	0.08	0.24	0.9987	93.30
Benzo(b)fluoranthene	5.478	0.10	0.30	0.9978	95.90
Benzo(k)fluoranthene	5.830	0.09	0.30	0.9985	85.20
Benzo(a)pyrene	6.162	0.07	0.21	0.9991	89.30
Dibenzo(a.h)anthracene	6.712	0.16	0.49	0.9946	78.20
Benzo(g,h,i)perylene	6.981	0.12	0.37	0.9969	72.80
Indeno(1.2.3-c.d) pyrene	7.165	0.07	0.23	0.9989	135.1

**Table 3:** Overview of the incidence, mean, minimum, and maximum levels of PAH12 in breast milk of Hungarian nursing mothers.

PAH	Number of Positive Samples	Mean (ng/mL)	Standard Deviation (ng/mL)	Minimum Detected Concentration (ng/mL)	Maximum Detected Concentration (ng/mL)	Sum (ng/mL)
Fluorene	26	5.34	10.52	0.50	46.08	266.91
Phenantrene	46	3.18	4.00	0.27	21.39	159.12
Anthracene	41	1.53	2.99	0.09	13.82	76.71
Pyrene	45	2.43	2.89	0.06	15.63	121.63
Benzo(a)anthracene	32	2.42	3.07	0.20	14.86	120.95
Chrysene	33	0.71	0.99	0.15	3.49	35.58
Benzo(b)fluoranthene	8	1.10	2.89	1.15	11.70	54.82
Benzo(k)fluoranthene	14	0.67	1.77	0.16	8.35	33.86
Benzo(a)pyrene	22	0.15	0.25	0.09	1.11	7.60
Dibenzo(a.h)anthracene	nd	nd	nd	nd	nd	nd
Benzo(g,h,i)perylene	18	0.17	0.28	0.16	1.43	8.38
Indeno(1.2.3-c.d) pyrene	18	0.15	0.27	0.00	1.43	7.74
ΣPAH (n = 50)						

**Table 4:** Demographic and health data of breastfeeding mothers.

Demographic Data of Participating Women (Number of Data)		Frequency (Percentage)
Location (n = 50)	Rural	18 (36%)
	Urban	32 (64%)
Education level (n = 47)	8 classes	5 (10.6%)
	skilled worker	8 (17%)
	maturity	14 (28%)
	graduated	20 (42.6%)
Marital status (n = 50)	in family	49 (98%)
	single	1 (2%)
Gravidity (n = 50)	First	27 (54%)
	Second	18 (36%)
	Third	5 (10%)
Ethnicity	Caucasian White	33 (82.5%)
	Caucasian Roman	3 (7.5%)
	Asian	3 (7.5%)
	Mixed race	1 (2.5%)
BMI (before the pregnancy)	Underweight	3 (6.3%)
	Normal	28 (58.3%)
	Overweight	10 (20.8%)
	Obese I.	3 (6.3%)
	Obese II.	3 (6.3%)
	Obese III.	1 (2.1%)
BMI (after the pregnancy)	Underweight	2 (4.2%)
	Normal	21 (43.8%)
	Overweight	11 (22.9%)
	Obese I.	9 (18.8%)
	Obese II.	2 (4.2%)
	Obese III.	3 (6.3%)

## Data Availability

The data presented in this study are available on request from the corresponding author, in order to protect the personal information of the mothers participating in the study.
